# Supplementary material for: The Effect of miR-140-5p with HDAC4 towards Growth and Differentiation Signaling of Chondrocytes in Thiram-Induced Tibial Dyschondroplasia
Source: Int J Mol Sci. 2023 Jun 30;24(13):10975. doi: 10.3390/ijms241310975 (PMC10342131; doi:10.3390/ijms241310975)
Supplement: Supplementary file 1 [file ijms-24-10975-s001.zip › ijms-2438819-supplementary.pdf]

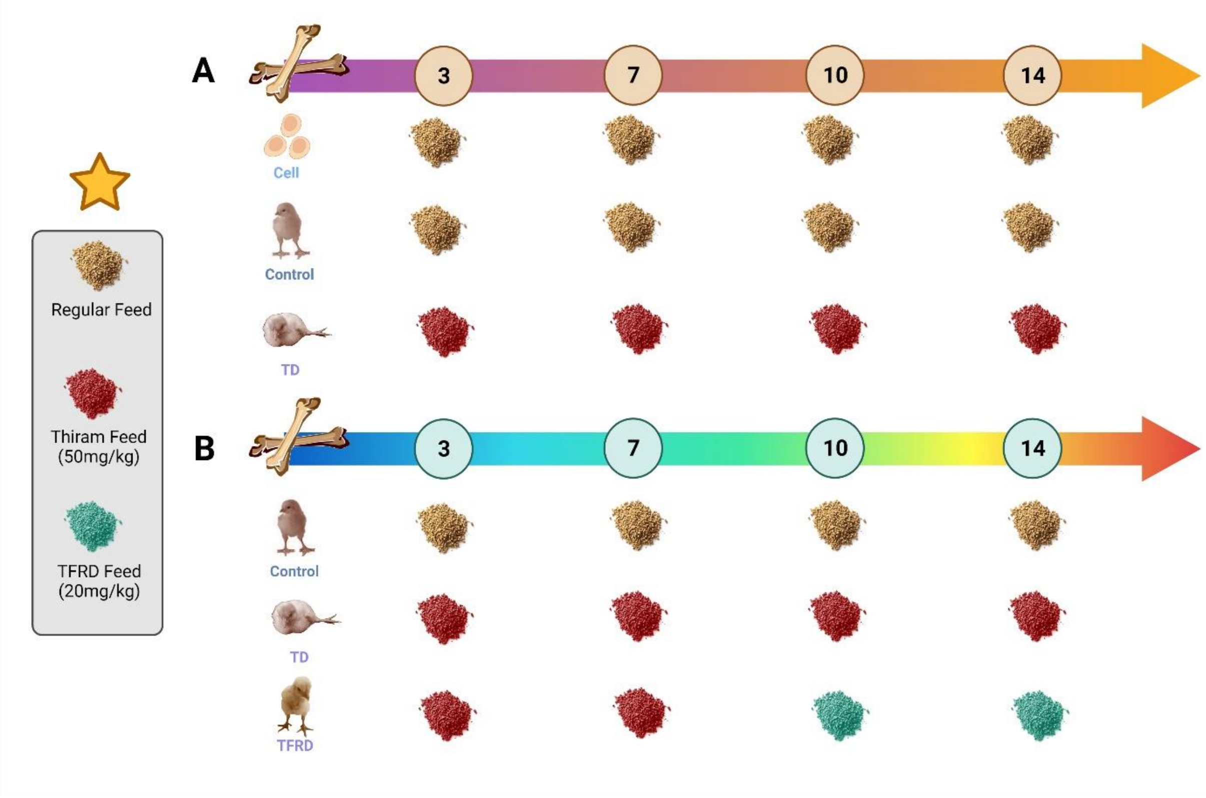

Supplementary Figure S1. A; The illustration for the culture of primary tibial growth plate chondrocytes and validation of miR-140-5p expression. **B;** The illustration of the *in vivo* experiment for administering total flavonoids of *Rhizoma Drynariae*.

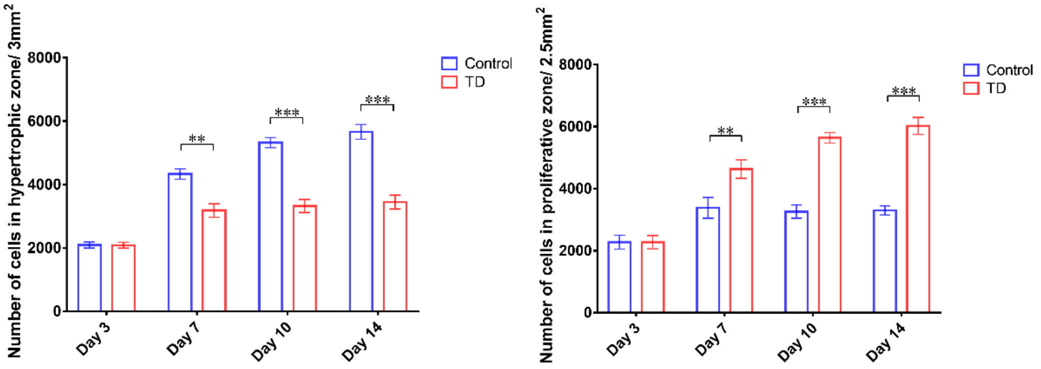

Supplementary Figure S2. The number of chondrocytes in hypertrophic and proliferative zones of control and TD groups on different days, i.e., 3, 7, 10, and 14. (** $p < 0.01$, *** $p < 0.001$).

A

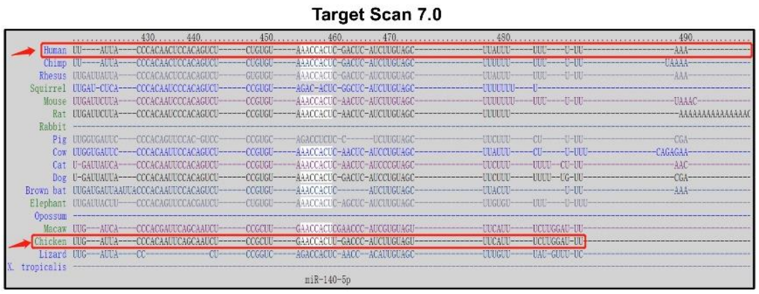

B

Names	Sequence	Length	Mass	Unused	Coverage (%)	Unique PepSeq	Peptide	Unique Peptide	Unique Spectrum	Spectrum	Unique Spectrum
Collagen alpha-1(X) chain OS=Gallus gallus OX=9031 GN=COL10A1 PE=4 SV=1	MHLQIS	674	66338.2	38.99	38.13	GEAGPV	19	19	1.1.1.778.	39	39
Collagen alpha-1(II) chain OS=Gallus gallus OX=9031 GN=COL2A1 PE=4 SV=1	MHGRRI	1420	134962.4	93.15	46.06	GPPGPQ	46	46	1.1.1.714.	96	96
Cartilage matrix protein OS=Gallus gallus OX=9031 GN=MATN1 PE=4 SV=1	MDGIFC	493	54047.6	32.13	31.03	SVRPQEI	16	16	1.1.1.835.	20	19

Supplementary Figure S3. A; Prediction of target relationship of miR-140-5p on the Targetscan7.2 website. **B;** Identification and evaluation of HDAC4-interacting proteins. After LC-MS/MS, the data were submitted to the Proteinpilot software connected to the AB Sciex Triple TOF™ 5600 plus mass spectrometer.

Supplementary Table S1 Primer used in this study.

Name	Accession number	Primer sequence (5′-3′)	Product size (bp)
**Col-II**	NM204426.1	F: ACCTACAGCGTCTTGGAGGA<br>R: ATATCCACGCCAAACTCCTG	155
**Sox9**	NM204281.2	F: GTCTCTGCCGGCTTTACTTCTTGT<br>R: TGCGAGAAAGCGGCACAGGG	113
**PTHrP**	AB175678.1	F: CGGAGGATATGATGTTCAC<br>R: TAGGAGGGCACAGAATAAC	79
**BMP-2**	XM015283435.1	F: TCAGCTCAGGCCGTTGTTAG<br>R: ACCCCACGTCATTGAAGTCC	185

**Runx2**	AF445419	F: TAAAGGTGACGGTGGATGG<br>R: TGTGGATTAAAAGGACTTGGTG	190
**Mef2c**	XM015280565.1	F: TTTGGGAATGAACAACCGTA<br>R: GGAAACCACTGGAGTAGCC	133
**Hdac4**	NM204313.2	F: TTAGTCACAGGACTGCCCCT<br>R: GTGCTGCTGCTGGATCACTA	165
**Col-X**	NM00103394.1	F: ATATCCACGCCAAACTCCTG<br>R:<br>TCAGAGGAATAGAGACCATTGGAT	471
**IHH**	NM204957.2	F: ACAGGGACCGCAACAAGT<br>R: CAGCCGAGTGCTCTGACT	122
**MMP13**	AF070478	F: CAACCCAAAACATCCCAAAAC<br>R: CCATTCATAGCCCAAACCTTC	258
**COMP**	XM040692880.1	F: CGAAGTCTTCTTGGAACAGCAG<br>R: AGACTTGTGCTGGGTTTGCT	230
**VEGF**	NM205042.2	F: CGAT GAGGGCCTAGAATGTGTC<br>R: AGCTCATG TGCGCTATGTGC	101
**GAPDH**	NM204305.1	F: GAACATCATCCCAGCGTCCA<br>R: CGGCAGGTCAGGTCAACAAC	137
